# 3D-Printed Microfluidic Droplet Generator with Hydrophilic and Hydrophobic Polymers

**DOI:** 10.3390/mi12010091

**Published:** 2021-01-16

**Authors:** Chandler A. Warr, Hunter S. Hinnen, Saroya Avery, Rebecca J. Cate, Gregory P. Nordin, William G. Pitt

**Affiliations:** 1 Department of Chemical Engineering, Brigham Young University, Provo, UT 84602, USA; chandlerwarr@gmail.com; 2 Department of Electrical Engineering, Brigham Young University, Provo, UT 84602, USA; hunterhinnen@gmail.com (H.S.H.); saroya_23@icloud.com (S.A.); beccacate@gmail.com (R.J.C.); nordin@byu.edu (G.P.N.)

**Keywords:** microfluidics, droplet generation, 3D printing, surface properties

## Abstract

Droplet generation has been widely used in conventional two-dimensional (2D) microfluidic devices, and has recently begun to be explored for 3D-printed droplet generators. A major challenge for 3D-printed devices is preventing water-in-oil droplets from sticking to the interior surfaces of the droplet generator when the device is not made from hydrophobic materials. In this study, two approaches were investigated and shown to successfully form droplets in 3D-printed microfluidic devices. First, several printing resin candidates were tested to evaluate their suitability for droplet formation and material properties. We determined that a hexanediol diacrylate/lauryl acrylate (HDDA/LA) resin forms a solid polymer that is sufficiently hydrophobic to prevent aqueous droplets (in a continuous oil flow) from attaching to the device walls. The second approach uses a fully 3D annular channel-in-channel geometry to form microfluidic droplets that do not contact channel walls, and thus, this geometry can be used with hydrophilic resins. Stable droplets were shown to form using the channel-in-channel geometry, and the droplet size and generation frequency for this geometry were explored for various flow rates for the continuous and dispersed phases.

## 1. Introduction

Microfluidic devices (MFDs) have emerged as highly versatile tools for a broad range of applications including biochemical analysis, biomedical assays and personalized medicine [1,2,3,4,5,6,7]. Integrating various unit operations such as droplet generation, optical analysis, electrochemical manipulation, pumping, and valves make MFDs poised for eventual use in the clinical setting. However, because MFDs are difficult to produce, they tend to fall into one of two manufacturing categories. The first includes high-resolution planar microfluidic devices (pMFD) [8,9,10,11], made the traditional way using stereolithography to make a negative mold, filling the mold with polydimethylsiloxane (PDMS) resin, and then covering the cured PDMS with glass to create fluid channels lying in the plane of the covering. This type of device allows for high-resolution channels well within the microfluidic regime (<100 μm) but confines them to a single plan, which ultimately limits crossing flows, which are needed for more complex flow patterns, multiple fluids, dynamic flow decision control, and other advanced unit operations.

The second category includes 3D-printed non-planar microfluidic devices (npMFD) [9,11,12,13] at size scales ranging from the large microfluidic (100–500 μm) regime to the lower-resolution millifluidic (>1 mm) regime. Such devices often have flow channels over and around each other in true 3D geometry. This category is summarized well by Beauchamp et al. [14], who assert that SLA -type 3D printers are currently the most practical printers for manufacturing npMFDs and that the smallest channels achievable in commercially available 3D printers are around 100 μm, which is still considered to be in the large microfluidic regime. This resolution limitation can present problems when attempting to manufacture a complex device that integrates multiple flows, valves, mixers, and two-phase flow (such as microdroplets). The limitations of these two categories of MFDs, high-resolution printing constrained to a single plane (pMFD) and the low-resolution printing of non-planar 3D microfluidic devices (npMFD), prompted the Nordin group to develop a high-resolution SLA printer and resin capable of manufacturing npMFDs with channel dimensions as small as 18 × 20 μm [15,16]. Subsequent publications based on our custom-built non-planar 3D printing system have demonstrated microchannels, pumps, control valves and mixers—all within a matrix of biocompatible polymers [17,18,19]. One unit operation yet to be demonstrated in our system is that of droplet generation, which has been shown to be key to many microfluidic operations [1,2,3,4,5,6].

Droplet generation has been demonstrated by many groups using pMFDs at small microfluidic sizes scale and npMFDs at large microfluidic and millifluidic size scales. For example, bacteria suspended in aqueous 50 μm droplets in an oil phase were demonstrated for a growth assay using a pMFD PDMS device with minimum channel dimensions of 10 × 20 μm [7], and a complex pMFD using a system of a Braille machine with actuated valves was shown for use in combinatorial drug screening using a minimum channel size of 50 × 50 μm in PDMS [1]. However, these pMFD devices have a fundamental limitation demonstrated by the above studies in that they are not capable of including all the necessary unit operations within the device because of the planar limitation. In other studies, complex double-emulsion droplets were formed using an npMFD from a polyjet 3D printer with a minimum channel size of 400 microns in the droplet-forming region [20], and a “plug-and-play” npMFD droplet generator system was developed with an SLA printer (Form 1+) using a minimum channel size of 262 μm [21]. However, these studies show that though 3D flow is achievable in npMFDs, it is not in the truly microfluidic regime and therefore could be improved and benefit from microfluidic channel sizes and smaller droplet volumes.

Because many droplet systems use aqueous droplets in continuous oil phases, the hydrophobicity of these npMFDs comes into play in that the channel walls must be sufficiently hydrophobic for the aqueous droplets, when encountering a wall, to bounce off instead of sticking. pMFDs using PDMS, which is very hydrophobic, have not been reported to accumulate aqueous droplets attached to walls. Kawakatsu et al. [22] comments that it is much more difficult to form water-in-oil droplets using a hydrophilic material than with a hydrophobic material because of the wetting that occurs on the channel walls, a problem other researchers have noted in their applications as well [1,20]. Therefore, in addition to the resolution limitation, npMFDs have a second obstacle to overcome, which is the wettability of the channel surface when making droplets. Though this problem has previously been addressed by post-processing to coat the surfaces of printed materials to generate the requisite hydrophobicity [23,24], a naturally hydrophobic resin eliminates the post-processing coating and facilitates the mass production of such npMFDs. For MFDs for non-biological applications, the requirements for the materials of construction are less problematic, and a sufficiently hydrophobic resin or post-processing surface treatment can be used. However, for MFDs used in biological applications such as cell growth, the materials of construction must not be toxic, or at least must be fairly biocompatible.

In our previous research making high-resolution npMFDs, we used a polyethylene-glycol (PEG) acrylate to provide the appropriate balance of biocompatibility, printability, and mechanical properties for control valves. However, the aqueous droplet generation in this previous system was problematic because the PEG-acrylate resin was not sufficiently hydrophobic. In this paper, we present two developments by which droplet generators can be made in high-resolution npMFDs on the custom printer that we previously developed and demonstrated [15,16,17,18,19,25]. We first developed and characterized a novel hydrophobic resin by measuring the hydrophobic/hydrophilic properties and correlating these with the resin formulation that allows the formation of aqueous droplets (in oil) with any desired droplet generator geometry. The second development is a truly 3D geometry for a microdroplet generator that allows consistent droplet formation using any of our hydrophilic or hydrophobic resins. This dual approach (material and geometry) provides two avenues for the successful and flexible implementation of microfluidic droplet generation in npMFDs.

## 2. Materials and Methods

### 2.1. Custom 3D Printer

Two 3D printers were employed in this study. The first is equipped with a Visitech LRS-WQ light engine having a 2560 × 1600 micromirror array and a 385 nm LED, whose wavelength requires the use of a resin containing a 2-nitrophenyl phenyl sulfide (NPS) UV absorber [15]. The build platform is a modified Solus 3D printing mechanism, holding a resin tray with a tensioned FEP (fluorinated ethylene propylene polymer) film. The other printer has an improved Visitech light engine with the same resolution (2560 × 1600 pixels) and a 365 nm LED, which permits the use of avobenzone as the UV absorber [19]. In addition, this printer has a 100 mm linear Griffin Motion stage with a single-axis Galil controller and a custom resin tray. Both printers use custom Python software that provides full control over all the printing parameters. With this custom code, the printing of each layer is carefully controlled, so as to make the exposure times and layer thickness for a 3D print easily adjustable throughout the experimental phases of resin development.

### 2.2. Materials

Custom resins were made following our previous procedure [15], wherein the resins were formulated with one or two acrylate monomers, a photoinitiator, and a UV absorber. The four different monomers investigated were trimethylolpropane ethoxylate triacrylate (TET), 1,6-hexanediol diacrylate (HDDA), lauryl acrylate (LA), and polyethylene glycol diacrylate (PEGDA). A PEGDA monomer containing Irgacure 819 photoinitiator (1%) and 2% NPS absorber was used in several recent publications [15,17,18] and served as a baseline comparison resin in this study. The above monomers were chosen to provide a monomer source for the final resin formulation with a variety of functional groups (mono-, di-, or tri-acrylates) and polarities (polar or non-polar). For example, mechanical stability (strength and stiffness) could most readily be imparted by the triacrylate TET monomer, but the monomer chemistry indicates that this formulation would be more polar and would tend toward producing a hydrophilic polymer. On the other hand, LA has only a single functional group, which tends to form a mechanically poor polymer, but would be very non-polar and could help to provide hydrophobicity to the final resin formulation. In addition to different monomers, two different UV absorbers were evaluated, corresponding to the 2 different light engines: 2% NPS for the 385 nm source, and 0.38% avobenzone (AVB) for the 365 nm source. The latter was developed as a biocompatible resin [19]. The particular concentrations of each absorber were chosen so that the optical penetration depth for each light source was essentially the same, namely, 12 μm, which facilitated our use of 10 μm-thick printing layers. At this optical penetration depth and photoinitiator concentration, the exposure times for these resins tend to be about 200–400 ms on our custom printer, though this depends on the light engine used. Our shorthand material designation for the printed materials is of the form ABS-MONOMER, e.g., A-HDDA (avobenzone UV absorber in HDDA monomer). See Table 1.

### 2.3. Contact-Angle Testing and Surface-Energy Calculation

The contact angles of seven fluids were measured on each of the materials polymerized from the resin formulations. Six of these fluids (water, octane, toluene, formamide, ethylene glycol, and nitromethane) were also used for surface-energy analysis. The seventh fluid, a fluorinated oil (Galden Perfluorinated Fluid HS 260), is often used as the continuous oil phase in our lab. The equipment used for the contact-angle measurement included a movable tilt stage, a diffuse light source, and a camera system. Freshly printed rectangular polymer pieces were cleaned with isopropyl alcohol (as per the normal protocol following the printing of these microfluidic devices) and then wiped with a Kimwipe to remove spurious polymer (or other) debris from the surface. The printed material was placed on the stage and brought into the focus of the camera. A single small droplet of fluid was placed on the top of the printed material, and a photograph was taken within 10 s. The picture was then analyzed with the ImageJ software to measure and report the contact angle. The surface energies were calculated using the Owens/Wendt method [26,27], with n ≥ 6 drops for each liquid, and the reported values are the mean ±95% confidence intervals.

### 2.4. Mechanical Hardness and Compression Testing

To characterize the mechanical properties of the resins, the hardness and compression modulus were measured for each resin. Measurements were made with 15 × 10 × 5 mm^3^ test chips, which were removed from the glass slides used for printing. Uni-axial compression tests were performed on each test chip using an Instron 3345 to provide the force-versus-compression-distance data from which the compression modulus could be calculated. The Shore D Hardness was measured for each resin type using a Rex Gauge Model 1600 durometer.

## 3. Results and Discussion

The objectives of this research were (1) to develop a resin system sufficiently hydrophobic for droplets to be able to generated in a pMFD, and (2) to develop a 3D npMFD droplet-generating geometry that made consistent droplets with any of our resins, hydrophobic or hydrophilic. The formulation of the resin, characterization of the material, and demonstration of the droplet formation in a pMFD are described in the first section of the Results and Discussion. After characterizing the polymerized resins, we explored various droplet-generation geometries printed with the hydrophilic PEGDA resin to determine if stable droplets could be generated using non-planar 3D geometries that are much more difficult to manufacture using traditional methods. The progress for different droplet-generator geometries, including a working version, and a characterization of the resultant droplet sizes and generation frequencies are presented in the later section.

### 3.1. Hydrophobic Resin

#### 3.1.1. Resin Formulation and Printing on Custom 3D Printer

The N-PEGDA and A-PEGDA resins used in previous publications [15,17,18,19] were used as baseline-comparison resins in this study, as they have been used for thousands of prints and worked well in the applications for which they have been used. This PEGDA resin has an optimized optical penetration of 12 μm set by the UV absorber and photoinitiator concentrations, and results in an optimal layer exposure time of around 240–270 ms using the 365 nm source. In order to test both polar and non-polar resin formulations, TET and HDDA were chosen as base monomers from which to construct new polar and non-polar resin formulas, respectively. The TET resin with the same optical penetration depth as the PEGDA resin needed an exposure time of around 200 ms using the 365 nm source, which resulted in a successful solid polymer. The HDDA resin under the same conditions needed an exposure time of around 350 ms to provide a solid polymer. The LA monomer never formed a solid polymer on its own but showed promise when added to the HDDA resin. By itself, the polymerized HDDA had some printing difficulties at times from adhering more strongly to the FEP film than did other monomers. It was found that a resin consisting of 85% HDDA and 15% LA (*w*/*w*) formed a solid polymer that printed well without the FEP adhesion problem; it also had very similar non-polar chemistry to HDDA and therefore did not compromise that property. This HDDA/LA resin at the same optical penetration and with the same 365 nm source required a layer exposure time of around 400 ms.

Therefore, out of the several tested resins, we decided to focus our efforts on the PEGDA, TET, HDDA, and HDDA/LA (85% HDDA-15% LA *w*/*w*) resins for material characterization and droplet generation, as these monomers produced good, reproducible polymers when printed and showed a range of polar and non-polar surface characteristics.

#### 3.1.2. Contact Angles and Surface Energy

**Figure 1:**
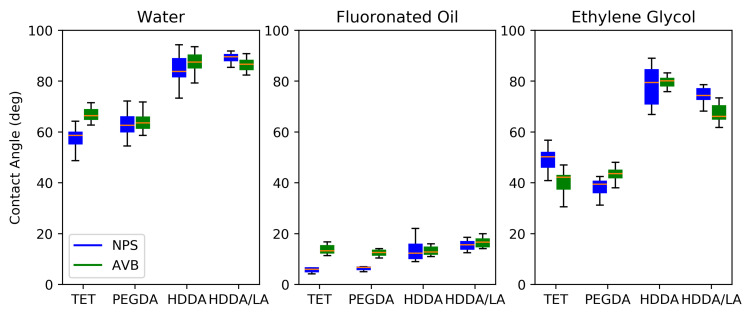
Contact angles for various fluids (water—polar; fluorinated oil—non-polar; ethylene glycol—mildly polar) and polymer combinations; n ≥ 6; error bars are 95% confidence intervals.

A polar and dispersive surface-energy analysis provides a useful characterization of and distinction between different polymers used for aqueous droplet generation. Hydrophilic polar polymers tend to attract polar fluids to the channel walls and not allow for the consistent formation and persistent stability of aqueous droplets. To determine the polar and dispersive (hydrophilic and hydrophobic) characters of the polymerized resins, the contact angles were measured and the associated surface energies calculated. The results are shown in Figure 1 for three of the tested fluids: water, fluorinated oil, and ethylene glycol. These three fluids show the behavior of the polymers when exposed to fluids of varying degrees of polarity: strongly polar water, mildly polar ethylene glycol, and non-polar fluorinated oil. The data show that different materials produce very different contact angles, particularly for water and ethylene glycol; however, the type of UV absorber produces very little difference in the contact angles. The water contact angle is most significant in evaluating a droplet-generation system, particularly when compared to fluorinated oil, which is used as the continuous fluid in many droplet applications. The chemistry of the individual monomers is consistent with the data obtained in the contact-angle analysis, given that the TET and PEGDA both have more available oxygen groups to allow for polar interactions with water, while HDDA and HDDA/LA have many more aliphatic hydrocarbon groups and fewer polar groups. For reference, the contact angle of water on a PTFE surface has been reported to be 113.7°, which is considered to indicate a very hydrophobic surface [28].

These contact-angle measurements were used to calculate the surface energies of the printed polymers according to the methods in [26,27], each of which illustrates the origins and use of the Owens/Wendt theory for calculating surface energy using a two-component model. This theory divides the total surface energy into polar and dispersive components. The model is shown in Equation (1), which includes the contact angle (θ) for the given fluid on the solid material as well as the overall, polar, and dispersive energies of the fluid ($\sigma_{L}$,$\sigma_{L}^{P}$,$\sigma_{L}^{D}$), which are reported in the references cited above.

(1) $$\frac{\sigma_{L}\left(\cos\theta +1\right)}{2\sqrt{\sigma_{L}^{D}}}=\sqrt{\sigma_{S}^{P}}\frac{\sqrt{\sigma_{L}^{P}}}{\sqrt{\sigma_{L}^{D}}}+\sqrt{\sigma_{S}^{D}}$$

From this model and our experimental contact-angle data, the polar and dispersive components of the solid material ($\sigma_{S}^{P}$,$\sigma_{S}^{D}$) were calculated. An example of such a calculation is graphically shown in Figure S1 in the supplemental material.

**Figure 2:**
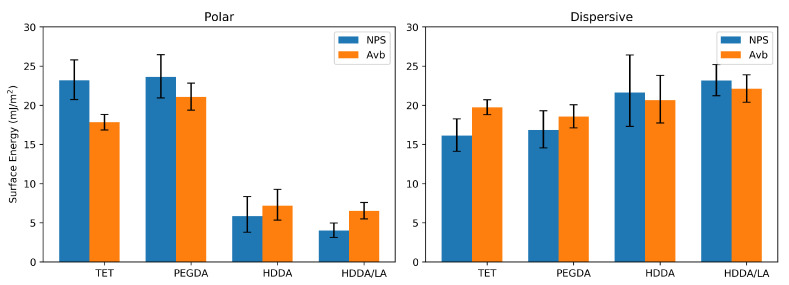
Polar and dispersive components of surface energy for various materials; n ≥ 6; error bars are 95% confidence intervals.

Figure 2 shows the polar and dispersive components of the surface energies of the various resins. Note that there is a significant difference in the polar surface-energy components for the different polymers, while there is not much variation in the dispersive components. HDDA and HDDA/LA both have much lower polar surface energies compared to PEGDA and TET regardless of the UV absorber. We therefore expect significantly different wetting for polar and non-polar liquids on the surfaces of 3D-printed features depending on which monomer or combination of monomers is used in the resin formulation.

#### 3.1.3. Mechanical Hardness and Compression Testing

**Figure 3:**
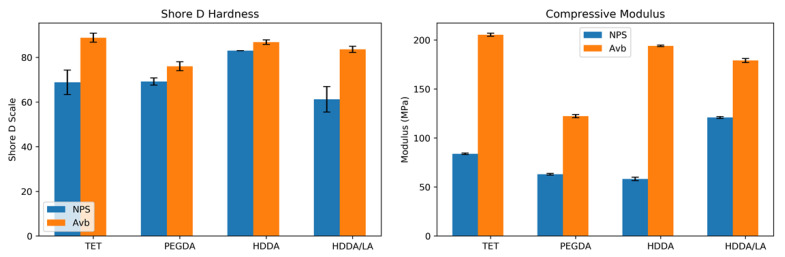
Shore D Hardness (n ≥ 5, 95% CI) and compression modulus (n ≥ 3, 95% CI) for various 3D-printed resin formulations.

We reported a preliminary evaluation of the mechanical properties of 3D-printed N-PEGDA resin in a previous publication [15]. Herein, we directly compare the compression modulus and hardness of our four monomer resin formulations for both the AVB and NPS 3D UV absorbers, as shown in Figure 3. Note that there is a clear difference in the compression modulus for resins with NPS and AVB UV absorbers, with the AVB UV abvsorber resulting in a substantially higher compression modulus for each monomer formulation. We consider it likely that this is due to the need for a much lower concentration of AVB (0.38%) compared to NPS (2%) to achieve the same optical penetration depth, which is the primary determinant of the *z*-resolution of a 3D print; i.e., a reduced concentration of small absorber molecules may facilitate a greater degree of crosslinking in the polymer matrix during 3D printing.

As seen in Figure 3, there is only a small difference in the hardness of 3D-printed resins when comparing the NPS and AVB UV absorbers. However, resins containing the AVB UV absorber were harder according to the Shore D scale than their NPS-containing counterparts, which is consistent with the increase in compression modulus.

In a previous publication, mechanical testing was performed with the N-PEGDA resin, and it was found that the Shore D Hardness was 75, a value within the margin of error in this study. However, the compression modulus found here is larger than the previously reported value [15]. One explanation for this may be the use of different custom printers and variations in the layer thicknesses of the prints themselves. There may also have been a difference in the optical curing time between the two studies; however, the previous study found that a post-curing UV exposure, which was also performed in this study, compensated for a lack of polymerization if the layer exposure time was not adequate.

#### 3.1.4. Droplet Formation

**Figure 4:**
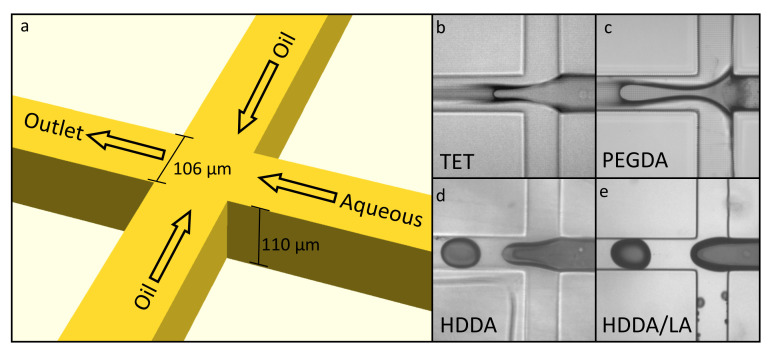
(**a**) 3D-printed microfluidic droplet generator. On the right (**b**–**e**) are shown simple pinch-off geometries built with A-TET, N-PEGDA, A-HDDA, and A-HDDA/LA. Oil flows were from top and bottom to the left. Channels were 106 μm across and 110 μm tall.

The initial test of the candidate resins’ abilities to enable droplet formation in 3D-printed microfluidic devices was evaluated using a simple cross-junction geometry (Figure 4a), which was a planar construct (pMFD). This geometry was built in OpenSCAD, where the rectangular channel was designed to be 106 μm (14 pixels) wide and 110 μm (11 layers) high. These were chosen to achieve roughly square microfluidic channels and were constrained by the 7.6 μm pixel pitch and 10 μm layer height generally used in our microfluidic printer. This method of fabricating microfluidic devices permits the realization of much more complex geometries than used here, but we chose this standard droplet-formation geometry to provide a simple scenario for droplet generation, and to evaluate which materials can form droplets in this planar geometry. Dyed deionized (DI) water and food-grade vegetable oil were used as the fluids in these tests, which were always run at the same flow rates of 5 μL/min.

Initial testing showed that the PEGDA resin does not produce consistent droplet formation; the fluid instead forms a co-flowing stream where the oil and aqueous phases both flow down the microfluidic channel, as shown in Figure 4c, with both phases in contact with the walls (the oil contacts the side walls and the water contacts the top and bottom walls). The other material candidates are shown as well, with TET (Figure 4b) having similar results to the PEGDA and forming co-flowing streams. On the other hand, the HDDA and HDDA/LA resins readily produced droplets under the same flow conditions (see Figure 4d,e).

It was observed that the UV absorber had little effect on droplet formation, as would be expected from the contact-angle and surface-energy data discussed earlier. As shown in Figure 4, it is clear that the HDDA resin was sufficiently hydrophobic to allow for droplet generation, but, as discussed earlier, the HDDA tended to adhere to the FEP film. Therefore, the LA monomer was added to HDDA, which greatly increased its printability while not compromising the hydrophobicity of the resin. It was observed that a resin consisting of 85% HDDA and 15% LA allowed for consistent droplet formation and did not compromise the material mechanical properties as observed for higher concentrations of LA, which is likely due to LA being monofunctional, thereby resulting in a less densely crosslinked polymer network. We therefore concluded, as demonstrated in Figure 4e, that in a practical sense, the HDDA/LA resin was the best candidate resin we investigated for the 3D printing of droplet-generating pMFDs. The size of the droplets generated and the generation frequency for this particular geometry were not investigated at this time because sufficient data are available for similar geometries from other researchers [29,30].

### 3.2. Hydrophilic Resin Geometry

#### 3.2.1. Immiscible Streams

While hydrophilic materials are problematic when attempting to form aqueous droplets [22], we theorized that a more sophisticated 3D droplet-generator geometry made possible by our high-resolution 3D printing technology may be able to circumvent these problems to consistently form stable droplets. Droplet formation is initiated when viscous forces overcome the surface energy and pull a droplet of the flowing stream off from the end of the flow [29,30]. Therefore, higher viscous forces produce a greater propensity for droplet formation. Traditional 2D droplet-generator geometries accomplish this by either surrounding and stretching the dispersed phase with the continuous phase, as in co-flowing or flow-focusing droplet-generator geometries [29,30,31], or by disrupting the dispersed phase with a perpendicular flow of the continuous phase, as in T-junction droplet-generator geometries [32,33].

**Figure 5:**
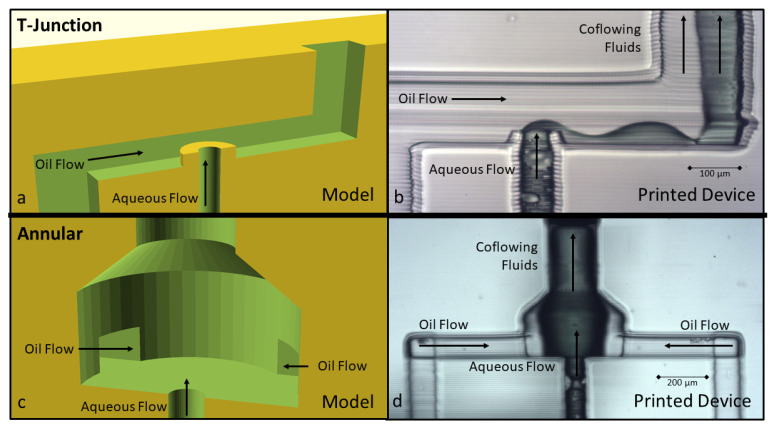
Cross section of the modeled and printed versions of 3D T-junction (**a**,**b**) and annular flow-focusing (**c**,**d**) geometries. Both geometries resulted in co-flowing streams where droplets did not form.

Using the A-PEGDA resin, we attempted to expand each of these traditional 2D geometries into analogous 3D versions as demonstrated in Figure 5. For example, Figure 5a shows a 3D T-junction geometry, and Figure 5b shows a photograph of the fabricated device, which reveals that the oil and aqueous streams co-flowed in the outlet channel. Even though this 3D T-junction geometry should impose more viscous forces by elevating the outlet of the dispersed phase above the bottom plane of the continuous-phase channel, the hydrophilic nature of the polymer allows the aqueous fluid to creep along the wall of the channel and form co-flowing phases instead of forming distinct dispersed-phase droplets.

Alternatively, Figure 5c,d show a flow-focusing geometry expanded into an annular 3D shape using the same hydrophilic resin. The continuous oil phase surrounds the outlet of the aqueous phase. Though the outlet water is, in theory, instantly surrounded on all sides by the oil phase, the aqueous phase still clings to the hydrophilic polymer and creeps along the wall of the channel instead of forming droplets. These examples illustrate the strength of the hydrophilic attachment between the aqueous phase and the polymer surface, and they suggest that a geometry that steers the viscous forces of the continuous phase towards droplet formation (instead of flow connected to the polymer wall) should be employed.

#### 3.2.2. 3D Annular Channel in Channel

**Figure 6:**
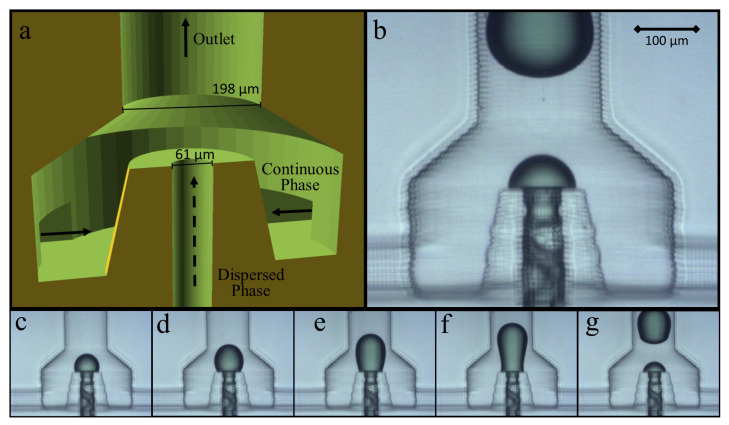
Modeled (**a**) and printed (**b**) 3D annular channel-in-channel (ACC) design, which allows for stable droplet formation in a hydrophilic A-PEGDA polymer. (**c**–**g**) show the progression of stable aqueous droplet formation.

A successful droplet generator made from the hydrophilic A-PEGDA polymer was developed by elevating the outlet of the dispersed phase in the annular flow-focusing geometry in Figure 5c (see Figure 6), referred to as an annular channel-in-channel (ACC) geometry. This ACC geometry directs viscous forces by limiting the possible contact area between the aqueous phase and the polymer after exit from the aqueous channel in the annular region at the top of the pedestal. Video S2 in the supplementary material shows stable droplets being formed using the ACC geometry. As Figure 6c–g show, the aqueous flow is still attached to the hydrophilic polymer at the raised outlet, and the droplet is formed by forcing the dispersed phase fluid to expand and disconnect from the flow channel without allowing for the dispersed (aqueous) phase to contact the hydrophilic polymer beyond the top of the annular pedestal. Using this geometry, droplets were formed that were consistent in size and stable through the downstream flow channels.

**Figure 7:**
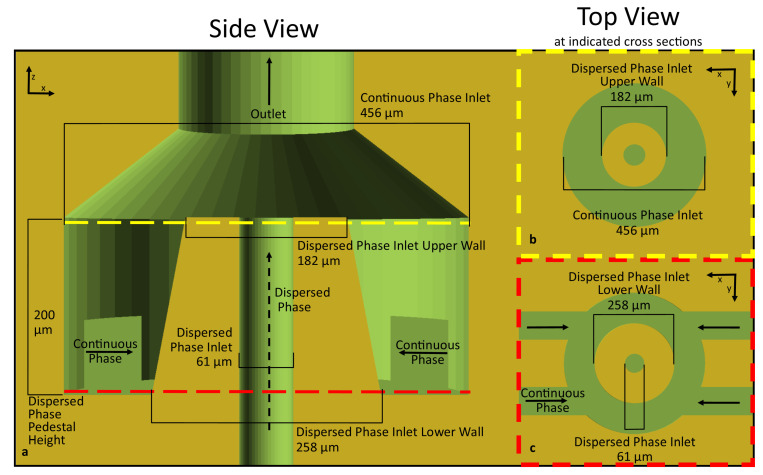
Cross sections and dimensions for annular channel-in-channel geometry shown in Figure 6. (**a**) shows an XZ cross section of the ACC geometry with the relevant dimensions and inlet locations of the dispersed and continuous phase flows. (**b**,**c**) show two different XY cross sections at upper (**b**) and lower (**c**) locations on the ACC droplet generator.

The geometry shown in Figure 6 is exemplary but not necessarily optimized. Variations on this geometry could be made by modifying the dimensional parameters and, in conjunction with the flow rates of the continuous and dispersed phases, be used to control droplet size and generation frequency. A pinch-off region could also be incorporated, similar to a flow-focusing geometry, which would provide for another parameter for controlling droplet size. Additional detail of the particular geometry shown in Figure 6 can be seen in Figure 7. Given the 7.6 μm pixel pitch of the custom printer used in this study, the geometry shown in Figure 6 does not necessarily utilize the highest resolution possible in our printer. The smallest channel dimension shown here is 61 μm, which is 8 pixels in diameter, while prior studies have shown that 3-pixel channels (23 μm) are possible [15]. Beyond the resolution of the printer, the dimension limiting this geometry may be the thickness of the annular wall making up the dispersed-phase outlet pedestal, though more research would need to be conducted to determine the ultimate limits of this ACC geometry. However, those limits will be very dependent on the resolution of the 3D printer as well as the combination of the resin formulation and printing parameters used.

#### 3.2.3. Droplet Size and Generation Rate

**Figure 8:**
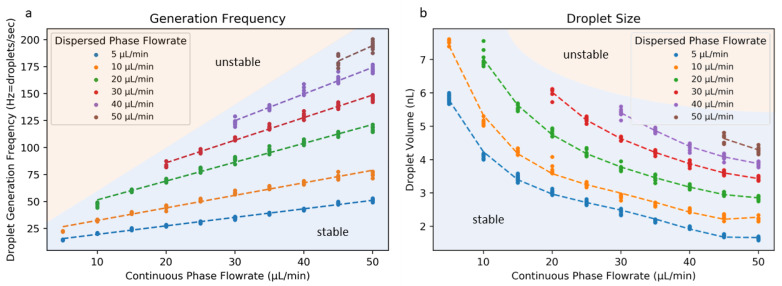
Generation frequency (**a**) and droplet size (**b**) associated with the ACC geometry for various flow rates for the dispersed and continuous phases. Flow rates for the continuous phase (vegetable oil) too far below those for the dispersed phase (water) resulted in unstable flow. Individual data points represent single experiments.

Using this ACC geometry, we investigated how various flow rates for the continuous and dispersed phases affected the droplet size and generation frequency. Figure 8 shows how variation in the continuous phase flow rate affects both the generation frequency and droplet size for several dispersed-phase flow rates. These data were generated using the ACC geometry of Figure 6 using the N-PEGDA resin. The generation rate shown in hertz (droplets formed per second) increased as the continuous-phase (oil) flow rate increased, and the droplet size decreased accordingly. This particular ACC geometry produced droplets in the 1–8 nL range while producing droplets at 10–200 Hz. For continuous-phase flow rates too far below the dispersed-phase flow rate, droplet generation becomes unstable, as the continuous phase does not produce the viscous shear forces necessary for dispersed-phase droplet formation. These data provide a basis for future work using this ACC geometry (particularly with hydrophilic resins) and other similar geometries that are possible through non-planar microfluidic 3D printing.

## 4. Conclusions

In this study, two approaches to successfully forming droplets in 3D-printed microfluidic devices were investigated. In the first approach, polar and non-polar resin candidates were investigated and evaluated for their suitability. The candidates included PEGDA, TET, HDDA, LA, and combinations thereof containing a photoinitiator and one of two possible UV absorbers. Significant differences in wettability between the resins were revealed by the contact angles of various fluids on polymers printed from these resins, corresponding to differences in surface-energy parameters. We found that the TET and PEGDA resins were too hydrophilic to produce stable aqueous droplets dispersed in oil using a basic planar cross-junction geometry. However, the use of HDDA and HDDA/LA resins produced materials sufficiently non-polar for aqueous droplets to consistently form that resisted attachment to channel walls, even in a planar MFD.

A second approach to forming droplets was investigated using the hydrophilic PEGDA resin with modified flow geometries. Standard 2D geometries were transformed to 3D versions for T-junctions and annular flow focusing, utilizing the full 3D design freedom afforded by 3D printing. In a first iteration, neither version successfully formed droplets. However, a further iteration consisting of an annular channel-in-channel design successfully formed stable aqueous droplets using the hydrophilic polymer.

In conclusion, this study showed that a HDDA/LA 3D printing resin was sufficiently hydrophobic to allow for droplet formation in 3D-printed planar microfluidic devices for the very basic geometries available in traditional microfluidic manufacturing. Additionally, a new non-planar 3D geometry was demonstrated that forms stable droplets using a hydrophilic PEGDA resin, thereby demonstrating that it is possible for 3D geometries to overcome the inherent disadvantages of hydrophilic materials for effective droplet generation.

## Figures and Tables

**Table 1:** Printing resin acronym codes.

Resin Code	Monomer	UV Absorber (*w*/*w*)	Photoinitiator (*w*/*w*)
N-PEGDA	polyethylene glycol diacrylate	2% NPS	1% Irgacure 819
A-PEGDA	polyethylene glycol diacrylate	0.38% Avo	1% Irgacure 819
N-TET	trimethylolpropane ethoxylate triacrylate	2% NPS	1% Irgacure 819
A-TET	trimethylolpropane ethoxylate triacrylate	0.38% Avo	1% Irgacure 819
N-HDDA	1,6-hexanediol diacrylate	2% NPS	1% Irgacure 819
A-HDDA	1,6-hexanediol diacrylate	0.38% Avo	1% Irgacure 819
N-HDDA/LA	85% 1,6-hexanediol diacrylate −15% lauryl acrylate	2% NPS	1% Irgacure 819
A-HDDA/LA	85% 1,6-hexanediol diacrylate −15% lauryl acrylate	0.38% Avo	1% Irgacure 819

## Data Availability

The data presented in this study are available on request from the corresponding author.

## Supplementary Materials

The following are available online at https://www.mdpi.com/2072-666X/12/1/91/s1: Figure S1: Graphical surface-energy calculation; Video S2: Channel-in-channel droplet formation.
